# Tracking macrophages in diabetic neuropathy with two-color nanoemulsions for near-infrared fluorescent imaging and microscopy

**DOI:** 10.1186/s12974-021-02365-y

**Published:** 2021-12-23

**Authors:** James M. Nichols, Caitlin V. Crelli, Lu Liu, Hoang Vu Pham, Jelena M. Janjic, Andrew J. Shepherd

**Affiliations:** 1 Division of Internal Medicine, Department of Symptom Research, The University of Texas MD Anderson Cancer Center, 6565 MD Anderson Blvd., Houston, TX 77030 USA; 2 School of Pharmacy, Duquesne University, 600 Forbes Ave., Pittsburgh, PA 15282 USA

**Keywords:** Diabetes, Neuropathy, Macrophage, Nanoemulsion, Skin, Sciatic nerve, Dorsal root ganglion, Spinal cord, Near-infrared fluorescence imaging, Neuroinflammation

## Abstract

**Background:**

The incidence of diabetes and diabetic peripheral neuropathy continues to rise, and studies have shown that macrophages play an important role in their pathogenesis. To date, macrophage tracking has largely been achieved using genetically-encoded fluorescent proteins. Here we present a novel two-color fluorescently labeled perfluorocarbon nanoemulsion (PFC-NE) designed to monitor phagocytic macrophages in diabetic neuropathy in vitro and in vivo using non-invasive near-infrared fluorescent (NIRF) imaging and fluorescence microscopy.

**Methods:**

Presented PFC-NEs were formulated with perfluorocarbon oil surrounded by hydrocarbon shell carrying two fluorescent dyes and stabilized with non-ionic surfactants. In vitro assessment of nanoemulsions was performed by measuring fluorescent signal stability, colloidal stability, and macrophage uptake and subsequent viability. The two-color PFC-NE was administered to Lepr^db/db^ and wild-type mice by tail vein injection, and in vivo tracking of the nanoemulsion was performed using both NIRF imaging and confocal microscopy to assess its biodistribution within phagocytic macrophages along the peripheral sensory apparatus of the hindlimb.

**Results:**

In vitro experiments show two-color PFC-NE demonstrated high fluorescent and colloidal stability, and that it was readily incorporated into RAW 264.7 macrophages. In vivo tracking revealed distribution of the two-color nanoemulsion to macrophages within most tissues of Lepr^db/db^ and wild-type mice which persisted for several weeks, however it did not cross the blood brain barrier. Reduced fluorescence was seen in sciatic nerves of both Lepr^db/db^ and wild-type mice, implying that the nanoemulsion may also have difficulty crossing an intact blood nerve barrier. Additionally, distribution of the nanoemulsion in Lepr^db/db^ mice was reduced in several tissues as compared to wild-type mice. This reduction in biodistribution appears to be caused by the increased number of adipose tissue macrophages in Lepr^db/db^ mice.

**Conclusions:**

The nanoemulsion in this study has the ability to identify phagocytic macrophages in the Lepr^db/db^ model using both NIRF imaging and fluorescence microscopy. Presented nanoemulsions have the potential for carrying lipophilic drugs and/or fluorescent dyes, and target inflammatory macrophages in diabetes. Therefore, we foresee these agents becoming a useful tool in both imaging inflammation and providing potential treatment in diabetic peripheral neuropathy.

## Background

In 2019 the International Diabetes Federation estimated the total number of adults with diabetes from age 20–79 was 463 million, with this number projected to increase to 578.4 million by 2030 [1]. Diabetic peripheral neuropathy (DPN) occurs in approximately 50% of people with diabetes and commonly presents as a distal symmetric polyneuropathy which begins in the distal limbs and progresses more proximally with time in what is known as a ‘stocking and glove’ pattern [2–4]. Patients with DPN may experience various symptoms in their arms and legs including numbness, tingling, pain and loss of thermal sensitivity, and can experience muscle weakness in the later stages of disease [2–5]. Unfortunately, current therapeutic options for DPN are limited in their ability to slow neuropathy or relieve pain. Analgesics used to treat DPN include tricyclic antidepressants, anticonvulsants, Selective serotonin reuptake inhibitors (SSRIs), serotonin and norepinephrine reuptake inhibitors (SNRIs), and opioids, which are only able to achieve greater than 50% pain relief in about one-third of patients [5].

Increasing attention is being given to the disruption of immune system activity in diabetes by hyperglycemia and/or chronic inflammation [6–8] particularly in adipose tissue in the case of obesity/type 2 diabetes [9]. These observations have profound implications for DPN, given the parallel emergence of the importance of neuroimmune crosstalk in neuronal dysfunction. One common immunological feature in different types of peripheral neuropathic pain is the contribution of peripheral macrophages and their cytokines, such as IL-6, IL-1β and TNF-α, to the sensitization of peripheral nerves [10, 11], orchestration of Schwann cell function [12] and clearance of damaged and dying cells [13]. Macrophages have been shown to be increased in the sciatic nerve of mice with high-fat diet-induced diabetes and rats with streptozotocin-induced diabetes [14, 15], and treatments which specifically target macrophages are able to reduce neuropathic pain in both models [14, 16]. Though evidence of macrophage contribution to DPN is now overwhelming, our mechanistic understanding of their contribution requires additional study. To that end, we have established a means of non-invasively tracking macrophages in Type 2 Diabetes Mellitus (T2DM), enabling longitudinal imaging to comprehensively assess disease progression and potential effects of future macrophage-directed therapeutics. We have chosen to target macrophages using the Lepr^db/db^ model of T2DM to determine if we can specifically image and track macrophage dynamics in this disease state, thereby obtaining unique insights into macrophage trafficking, phenotype and function.

Lepr^db/db^ mice have a single point mutation in the gene coding for the leptin receptor, which induces hyperphagia and obesity [17]. We chose this strain because it exhibits many of the metabolic characteristics seen in patients with T2DM including dyslipidemia, insulin resistance and hyperglycemia [17]. They also exhibit a significant loss of intraepidermal nerve fibers (IENF) over time, which are associated with the progressive loss of sensation in the distal limbs and is a common pathological finding in T2DM patients [4, 5, 17]. Given the similarities between Lepr^db/db^ mice and patients with T2DM, this model represents a strong candidate for examining new therapeutic options for DPN.

Macrophages are well established cellular targets for in vivo tracking by near infra-red fluorescent (NIRF) imaging and magnetic resonance imaging (MRI) [18–22] using multimodal perfluorocarbon nanoemulsions (PFC-NEs). PFC-NEs have been successfully used to non-invasively image distribution patterns of infiltrating macrophages in rodent transplant models (kidney, heart) and such PFC-NE labeled macrophage infiltration correlates well with histopathologic findings of rejection [19]. In a related study, PFC-NEs were also used to quantify macrophage infiltration as a surrogate measure of inflammation in pig hearts in a myocardial infarction model [23]. PFCs are chemically and biologically inert Fluorine-19 nuclear magnetic resonance (^19^F MRI) agents which allow for quantitative and qualitative assessment of inflammation in vivo following organ rejection [19], abscess formation [24] and inflammatory bowel disease [25]. By their physicochemical properties PFCs are liquids at ambient temperature, and immiscible with water or hydrocarbon oils. We have recently developed triphasic macrophage-targeted PFC-NEs, which are composed of two immiscible liquids: PFC (fluorous phase) and hydrocarbon oil (HC, organic phase), and stabilized with non-ionic surfactants in water (aqueous phase) [26–29].

The advantage of such triphasic PFC-NEs lies in their increased capacity to incorporate lipophilic drugs [26] and/or NIRF dyes, due to the higher organic volume fraction compared to other reported PFC-NEs, with no significant change in droplet size [30–34]. The NIRF labeled PFC-NEs show an excellent correlation between NIRF signal and ^19^F NMR signal in cells and tissues ex vivo [35]. PFC-NE were also used to image macrophages associated with neuroinflammation in rat nerve injury models [27, 36–38] and Complete Freund’s Adjuvant (CFA) induced inflammation model in mice [28, 39] by in vivo and ex vivo NIRF imaging. In these models, presence of PFC-NE labeled macrophages in target tissues was confirmed using immunohistochemistry and immunofluorescence microscopy [27, 28, 36, 38, 39]. Based on these findings it was conceivable that PFC-NEs with some adaptations could be used to track macrophages in DPN.

In this study we formulated a novel two-color fluorescently labeled PFC-NE designed to specifically label phagocytic macrophages in vitro and in vivo in DPN*.* The presented PFC-NEs enable specific tracking of the monocyte derived macrophages in the peripheral immune system using both live NIRF imaging systems and fluorescent microscopy in excised tissues. Here, for the first time to the best of our knowledge, the PFC-NE based system is applied to tracking macrophages along the length of the primary neurons of the peripheral sensory pathway in the hind limb of Lepr^db/db^ and WT mice combining live and ex vivo NIRF imaging, and fluorescent microscopy. The presented work establishes a new approach to investigating macrophages in DPN pathology and sets forth a path towards designing novel diagnostic and treatment assessment strategies for DPN that exploit neuroimmune crosstalk.

## Methods

### Mice

Experiments performed were approved by the MD Anderson Institutional Animal Care and Use Committee. Male Lepr^db/db^ and Lepr^WT/WT^(WT) (BKS.Cg-Dock7m + / + Leprdb/J) mice were obtained from Jackson Laboratories (Bar Harbor, ME) at approximately at 4 weeks of age for time course studies, or 11 weeks of age for histological studies, and allowed to acclimate to our animal facilities for > 1 week before experiments. Mice were housed two to five per cage in a temperature (22 °C ± 1 °C), and humidity (40–60%) controlled room. The room was also kept on a 12-h light dark cycle, and mice had access to food and water ad libitum.

### Two—color fluorescent PFC-NEs

PFC-NEs for in vivo and ex vivo fluorescent tracking of monocyte derived macrophages used in this study were prepared by adapting earlier published methods [27, 28]. PFC-NEs were formulated to incorporate two fluorescent dyes in the hydrocarbon phase. Perfluoro-15-crown-5 ether (14.2% w/v) was blended by vortexing with Miglyol 812 N (7.6% w/v) which incorporated two dyes: 1,1′-Dioctadecyl-3,3,3′,3′-Tetramethylindocarbocyanine Perchlorate [‘DiI’; DiIC18(3)], at 10 μM and (‘DiR’; 1,1′-Dioctadecyl-3,3,3′,3′-Tetramethylindotricarbocyanine Iodide, DiIC18(7)), at 10 or 25 μM final concentration, and with fluorescence emission maxima at 570 nm and 780 nm respectively. The oils were mixed with non-ionic surfactant solution prepared in 1X phosphate buffered saline (PBS) and processed on Microfluidizer M110S (Microfluidics, Westwood, MA 02090, USA) at 18,000 psi liquid pressure on 25 mL scale as reported earlier [27]. All nanoemulsions are sterilized by filtration through a 0.22 μm filter and stored in sterilized amber vials before use.

### Nanoemulsion fluorescent stability

All fluorescently labeled nanoemulsions were monitored for fluorescence signal stability upon storage at 4 °C using a LiCOR Odyssey imager. Briefly, nanoemulsion serial dilutions (e.g., 1:10, 1:20, 1:40, 1:80, 1:160) were prepared in deionized water and imaged in triplicate in 96-well clear flat bottom microplates at 800 nm fluorescence detection. The imaging parameters were kept constant, and measurements were repeated over time to establish overall fluorescence signal stability upon storage.

### Nanoemulsion colloidal stability

All nanoemulsions were monitored for size and size distribution changes over time and under varied conditions (centrifugation, filtration, and exposure to cell culture media at 37 °C), as earlier reported [27], by Dynamic Light Scattering (DLS) on Zeta Sizer Nano ZS (Malvern Instruments, Worcestershire, United Kingdom) instrument equipped with a HeNe gas laser (λ = 633 nm) and a detector at 173°. The nanoemulsion samples were diluted in deionized water or media at 1:40 v/v and all measurements carried out at 25 °C.

### Macrophage viability upon nanoemulsion exposure

Cell viability was evaluated with mouse macrophages (RAW 264.7; ATCC TIB 71) using the CellTiter-Glo^®^ 2.0 assay. Macrophages were plated in a 96 well plate at 5000 cells/well. After incubation overnight at 37 °C and 5% CO_2_, the cells were treated with dilutions of the 10 and 25 μM DiR PBS-based PFC-NE, starting at a maximum dilution of 80 µL of PFC-NE in 1 mL culture medium and following serial dilutions down to 0.625 µL per 1 mL. The cells were treated with these dilutions for 24 h at 37 °C and 5% CO_2_. Then, the media was removed from all wells and 100 µL fresh culture media was added to each well and 40 µL CellTiter-Glo^®^ 2.0 was added. The plate was shaken for 20 min at 70 rpm and protected from light to induce cell lysis. 100 µL of the cell lysate was transferred to a 96 well white plate and luminescence was recorded using the Synergy HTX Multi-mode reader from BioTek. Cell viability results can be seen in Fig. 3D.

### Macrophage microscopy upon nanoemulsion exposure

Images of mouse macrophages labeled with nanoemulsion were acquired using the EVOS™ FL Digital Inverted Fluorescence Microscope from Invitrogen. RAW 264.7 macrophages were cultured on glass chamber slides (Lab Tek Chamber Slides, 8 well slides) at 50,000 cells/well and incubated for 24 h. Macrophages were treated with nanoemulsion containing 25 µM DiR and 10 µM DiI at a dilution of 10 µL nanoemulsion in 1 mL of culture medium and were incubated for 24 h at 37 °C and 5% CO_2_. After removing the treatment media, macrophages were fixed with 0.3 mL 4% paraformaldehyde (PFA) in PBS for 10 min. The PFA was then removed and the macrophages were washed 3 times with PBS. The chambers were removed and a cover slip was mounted onto the slide using a mountant containing DAPI (ProLong™ Diamond Antifade Mountant with DAPI, Molecular Probes). Images were captured using the EVOS Microscope using the white light, DAPI, and Red Fluorescent channels. For visualizing, the DAPI channel, Invitrogen™ EVOS™ Light Cube, DAPI was used (excitation of 357/44 and emission of 447/60). For visualizing nanoemulsion uptake into the macrophages, Invitrogen™ EVOS™ Light Cube, RFP was used (excitation 531/40 and emission 593/40). Images of mouse macrophages treated with nanoemulsion can be seen in Fig. 3A–C.

### In vivo NIRF imaging

Images were taken of WT (*n* = 5) and Lepr^db/db^ (*n* = 5) mice with the IVIS Lumina system (Caliper Life Sciences; Waltham, MA) using the auto exposure setting (≤ 40 s.). Fluorescence from the DiR component of the nanoemulsion was detected with a 745 nm (bandpass: 20 nm) excitation and 800 nm (bandpass: 35 nm) emissions filter. After obtaining images from each mouse, regions of interest were drawn around each foot pad and the Average Radiant Efficiency ([p/s/cm^2^/sr]/[µW/cm^2^]) was obtained for each foot. The two feet were then averaged together to obtain an Average Radiant Efficiency for each mouse. Mice were imaged weekly from 7 weeks of age to 12 weeks of age. At 8 weeks of age, 200 µl of PFC-NE was injected intravenously (i.v.) into the tail vein of each mouse. The mice were allowed to rest for 72 h prior to imaging for the 8-week time point.

### Perfusion and tissue processing

**Fig. 1:**
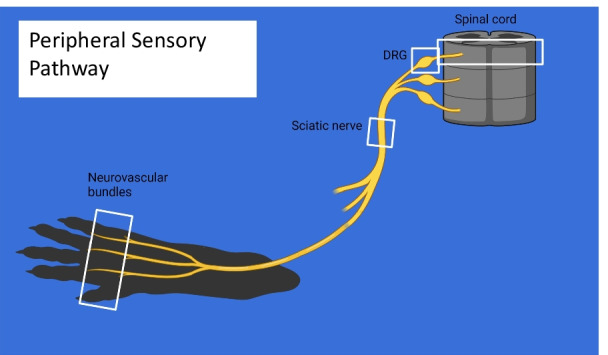
Peripheral sensory pathway. To accurately assess our ability to target macrophages at multiple points along the primary neurons of the peripheral sensory pathway, microscopic examination was performed on tissue from hind paws, thighs (encompassing the sciatic nerve trunk) and spinal column to carry out a survey of CD68^+^DiI^+^ macrophages in the neurovascular bundles, sciatic nerve and spinal cord/DRGs, respectively

To determining the tissue distribution of the PFC-NE, 12-week-old WT (*n* = 3) and Lepr^db/db^ (*n* = 3) mice were injected with 200 µl of PFC-NE. The PFC-NE was allowed to distribute throughout the tissues for 72 h prior to the mice being euthanized for histological analysis. At necropsy, mice were euthanized using CO_2_, and perfused with 20 mLs of cold PBS followed by 20 mLs of cold 10% neutral buffered formalin (10% NBF) (Thermo Scientific; Waltham, MA). Feet, thighs, and spinal columns were obtained from each mouse to assess the neurovascular bundles of the feet, sciatic nerves and DRGs/spinal cords respectively, as these represent the various levels of the of the primary neurons of the peripheral sensory pathway (Fig. 1). These tissues were submerged overnight at 4 °C in 10% NBF before further processing. To decalcify the tissues, they were placed in PBS containing 10% EDTA (Sigma-Aldrich; St. Louis, MO) for 14 days at 4 °C, and the solution was refreshed after 7 days. Once decalcification was complete, the tissues were placed in 30% sucrose for two days at 4 °C, and subsequently embedded in Tissue-Tek^®^ OCT compound (Sakura; Torrance, CA) for cryosectioning.

### Tissue cryosectioning

For the purpose of our histologic survey, each tissue was sectioned at 30 µm and placed on Superfrost Plus slides (Fisher Scientific; Pittsburgh, PA). The sections were allowed to adhere to the slides at room temperature (RT) for approximately 10 min prior to being placed at – 20 °C, since prolonged exposure to RT tended to reduce the DiI signal within the tissues. The slides were kept at – 20 °C until they were stained.

Transverse sections of both the left and right feet of each mouse were obtained at the distal end of metatarsals 2–4. This allowed images to be taken of the neurovascular bundles present between the 2nd, 3rd, and 4th metatarsals as well as survey the entire foot for distribution of PFC-NE. For the sciatic nerve, transverse sections of the left and right thigh of each mouse were obtained at the proximal end of the femur so that the sciatic nerve could be visualized along with the surrounding tissue.

While embedding the spinal column, care was taken to isolate the sections of the spinal column which contained the lumbar spinal cord, and the lumbar dorsal root ganglia (DRG). Based on a study by Harrison et al. [40] it was determined that the lumbar spinal cord could be obtained by focusing our sectioning between the 10^th^ thoracic vertebra (T10) and halfway through the 1^st^ lumbar vertebrae (L1). This allowed us to separate the parts of the spinal column containing the lumbar spinal cord from the part containing the lumbar DRGs (L1–L6) and section them separately.

### Immunofluorescence

Sections were removed from -20˚C and allowed to warm to RT. During this time a hydrophobic barrier was drawn on each slide using an ImmEdge™ Pen (Vector Laboratories; Burlingame, CA). Slides were dipped 10 × in PBS to remove residual OCT before the adding PBS containing 10% goat serum (Sigma)/1% Bovine serum albumin (Fisher Scientific)/ 2% DMSO (Sigma)/ 1 mg/mL digitonin (Millipore; Burlington, MA) (Blocking solution). Digitonin was used as a detergent since it has previously been shown to be superior to other detergents for the retention of DiI [41]. The slides were incubated in blocking solution for 1 h at RT. The blocking solution was replaced with antibodies diluted in blocking solution: rabbit anti-PGP9.5 (1:200) (Abcam; Clone: EPR4118) and rat anti-CD68 (1:500) (Biorad; Clone: FA-11) were both added to the slides in blocking solution. Primary antibodies were incubated overnight for at 4 °C in a humidified slide staining chamber. The next day the slides were rinsed by dipping 10 × in PBS, and goat anti-rabbit Alexa Fluor 647 (1:500; Invitrogen; Carlsbad, CA), goat anti-rat Alexa Fluor 488 (1:500; Invitrogen), and DAPI (500 ng/mL; Sigma) were added to each slide in PBS. The secondary antibodies and DAPI were incubated for 3 h at RT. Slides were dipped 10 × in PBS, and 10% NBF was added to the sides for 10 min to help maintain DiI signal within the tissues. The sections were rinsed one final time by dipping 10 × in PBS, and coverslipped with Prolong Gold Mounting Medium (Cell Signaling Technology, Danvers, MA). For the purpose of setting thresholds for analysis, control negative stains were generated for CD68 and PGP9.5 by omitting the primary antibodies. Additionally, DiI signal was verified by dipping a slide in PBS 10x, fixing the slide with 10% NBF, and coverslipping with Prolong Gold as described above (Fig. 5).

### Confocal microscopy and image quantification

Tile scans of tissue sections were obtained using 20x (Numerical aperture: 0.75) objective on a Nikon A1R Confocal Microscope (Nikon Instruments Inc., Melville, NY), and Image analysis was performed using Nikon NIS-Elements Advanced Research (Nikon Instruments Inc.) These images were then used to determine the tissue distribution of the i.v. injected PFC-NE within the foot, thigh and spinal cord of WT (*n* = 3) and Lepr^db/db^ (*n* = 3) mice (Table 1).

**Table 1:** Summary of tissues containing CD68 + NE + double positive cells.

Tissue	CD68 + cell location	WT	Leprdb
Hind Foot	Dermis	+	+
	Hypodermis	+	+
	Bone marrow (Metatarsals)	+	+
	Nerves	−	−
	Adipose tissue	+	+
Thigh	Bone marrow (Femur)	+	+
	Perineural adipose tissue	+	+
	Muscle	+	+
	Sciatic nerve	+ (weak)	+ (weak)
Lumbar DRG	DRG	+	+
	Perineural adipose tissue	+	+
	Meninges	+	+
Lumbar spinal cord	White matter	−	−
	Gray matter	−	−
	Meninges	+	+
	Bone marrow (Vertebra)	+	+
	Nerve roots	−	−

Summary of the distribution of CD68^+^NE^+^ double positive cells within the various tissues collected during this study. + present, − absent

To quantify the distribution of DiI from PFC-NE within tissue macrophages surrounding the neurovascular bundles of the foot pads from WT and Lepr^db/db^ mice, measurements for percent area of CD68 and DiI, and Pearson’s correlations between CD68 and DiI were determined for the hypodermal area surrounding the neurovascular bundles between metatarsals 2–4 in both the left and right foot (four per mouse). For this analysis a “region of interest” (ROI) was drawn around each hypodermal region (Fig. 7) and a threshold was applied to the CD68 and DiI signals in this region to measure the percent areas for each signal. To assess differences between the uptake of PFC-NE within the sciatic nerves of WT and Lepr^db/db^ mice and peripheral tissues immediately adjacent to the sciatic nerves (muscle and adipose), individual ROIs were drawn around three CD68^+^DiI^+^ double positive cells from the sciatic nerve and peripheral tissue on each slide for measurement mean DiI intensity. The cellular intensities were then averaged for each tissue, and statistical analysis was performed on these averages. To assess signal intensity differences within DRG tissues between WT and Lepr^db/db^, the mean signal intensities were measured for a lumbar DRG from each mouse and differences between groups were analyzed. A ROI was drawn around the neuronal cell bodies within the DRG (Fig. 11) and a threshold was applied to the DiI signals in ROIs to measure the mean intensity of cells containing DiI.

### Statistical analysis

For each data set Gaussian distribution was determined using a Shapiro–Wilk test and based on these results appropriate parametric and non-parametric statistical analysis was performed. A post-hoc power analysis was also run on each data set to determine that sufficient statistical power was achieved. Unpaired nested *T*-tests were performed on the percent area calculations for DiI and CD68 and Pearson’s correlations from the histological sections of the foot. An unpaired *T*-Test was performed on the DiI signal intensity from DRG images. Two-way ANOVA was performed on average radiant efficiencies from NIRF images, and Wilcox matched-pairs signed rank tests were performed for the comparison of sciatic nerve tissues to peripheral tissues. Weight corrections for average radiant efficiencies were performed by multiplying the average radiant efficiency for each animal by the weight of the animal and then normalizing to average baseline reading for the group (week 7).

## Results

### Two-color fluorescent perfluorocarbon nanoemulsion (PFC-NE)

**Fig. 2:**
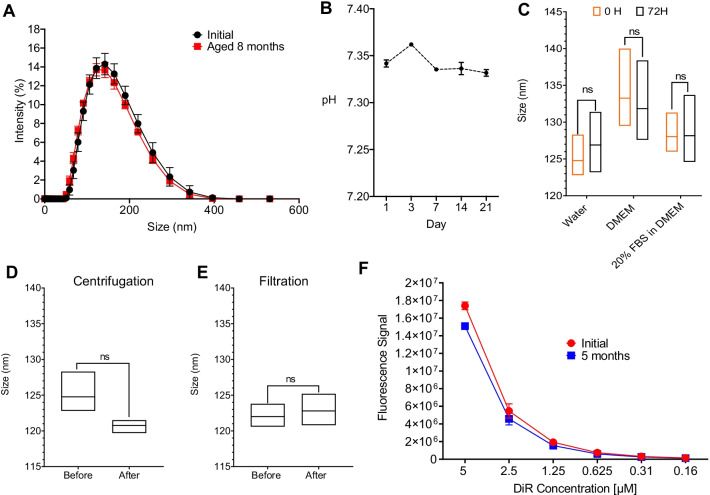
Macrophage targeted two-color (DiI/DiR) fluorescent nanoemulsion. **A** PBS-based PFC-NE particle size distribution overlay comparison between day 1 and 8 months after manufacturing. **B** pH stability of PFC-NE measured upon manufacture and up to 21 days. **C** PFC-NE serum stability in three different biological media (Water, DMEM, and 20% FBS in DMEM) was measured at time 0 and after 72 h incubation at elevated temperature of 37 °C (*p* = 0.514, *p* = 0.777, *p* = 0.969 respectively, Student’s *T*-test, *n* = 3). **D** PFC-NE centrifugation stability was conducted with 3000 rpm for 30 min. PFC-NE (undiluted) size was measured before and after centrifugation (*p* = 0.097, Student’s T-test, for *n* = 3). **E** PFC-NE stability was tested and analyzed before and after sterile-filtration with a 0.22 μm MCE membrane filter. All particle size and/or distribution were measured by Dynamic light scattering (DLS) (*p* = 0.642, Student’s *T*-test, *n* = 3). **F** Fluorescence signals of the PFC-NE with NIRF dye DiR were compared between week 1 and week 20 after manufacturing. PFC-NE was diluted at PFC-NE to water ratio of 1:4. The fluorescence signal was collected by LI-COR Odyssey Imager. Error bar represents the standard deviation from three independent measurements

**Fig. 3:**
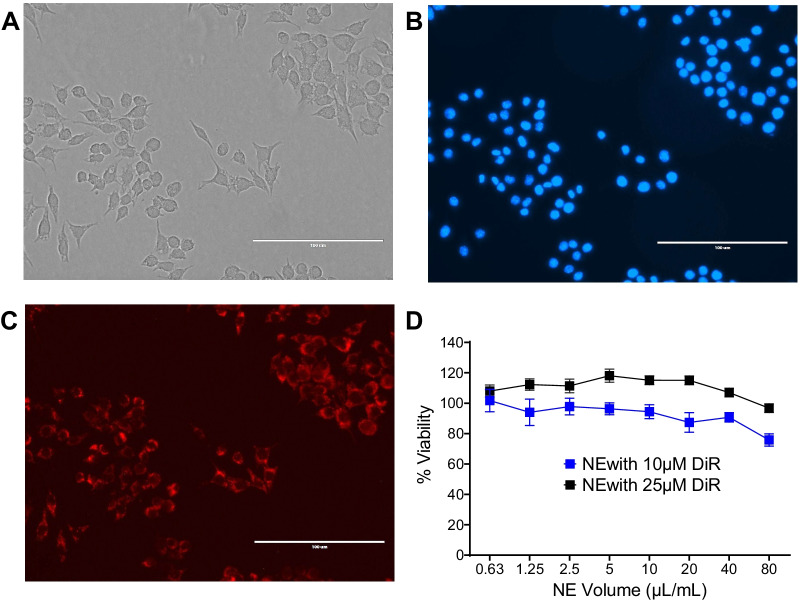
Nanoemulsion uptake and impact on macrophage viability in vitro. PBS-based PFC-NE uptake when exposed to RAW 264.7 (ATCC TIB 71) macrophage cell line. Microscopy images were taken on EVOS Imager at 40X Magnification. White bar on the lower right corner represents 100 µm **A** Image of macrophage morphology when exposed to PFC-NE under the White Light channel. **B** Image of macrophages’ nuclei when exposed to PFC-NE, and treated with Hoechst stain under the DAPI channel. Cell nucleus can be observed in blue. **C** Image of macrophages’ cytoplasm when exposed to NE under the Red Fluorescent Channel. **D** Macrophage cell viability upon 24 h exposure of NE with 10 µM and 25 µM of Near infrared (NIRF) dye DiR concentration. Cellular viability was assessed using CellTiter-Glo^®^ 2.0. The error bar represents the standard deviation from six independent measurements

In DPN models, we required PFC-NE fluorescence to maintain signal for long term follow-up by in vivo and ex vivo imaging, and provide macrophage-specific intracellular fluorescent labeling in excised tissues. Introduction of fluorescent dyes to PFC-NEs have proven challenging with reports of fluorescence dissociation from the nanoemulsion droplets [42]. However, our group has demonstrated that triphasic PFC-NEs can incorporate NIRF dyes into the hydrocarbon shell, and maintain fluorescence signal stability upon storage and use [29]. We also show that fluorescently labeled macrophages can be detected in tissues 40 days post PFC-NE intravenous injection at the site of inflammation by immunofluorescence [39]. In related studies we also showed successful fluorescent labeling of PFC-NEs which resulted in long term tracking of PFC-NE labeled macrophages in varied animal models [27, 28, 35–39, 43]. For the purpose of macrophage tracking in DPN model with NIRF imaging and immunofluorescence we formulated a novel two-color fluorescently labeled perfluoro-15-crown-5 nanoemulsion with two distinct lipid tracers, DiI and DiR, incorporated into the hydrocarbon phase at optimized ratio 1:2.5 w/w. DiI with fluorescence maximum at 570 nm is used for detection of nanoemulsion labeled macrophages in excised tissues by fluorescent microscopy, while DiR supports live and ex vivo NIRF imaging of macrophage infiltration in target organs and tissues in DPN mice. This dual labeling of PFC-NEs proved stable as measured by fluorescence over time for up to 5 months (Fig. 2F). Nanoemulsions were produced in phosphate buffer to assure biocompatibility and safety in animals upon i.v. injection. Dual labeled nanoemulsions show exceptional colloidal stability upon prolonged storage (Fig. 2A), exposure to biological media and stress (Fig. 2B–E) and also demonstrated long term fluorescence stability (Fig. 2F) upon storage for 5 months. Furthermore, presented nanoemulsions effectively labeled macrophages in culture upon 24 h exposure (Fig. 3A–C) and caused no significant change in viability over a range of nanoemulsion concentrations (0–80 µL/mL). High fluorescence and colloidal stability are essential for maintaining consistent in vitro and in vivo NIRF imaging-based macrophage tracking which is critical for monitoring dynamic changes in inflammation in DPN animals.

### NIRF imaging

**Fig. 4:**
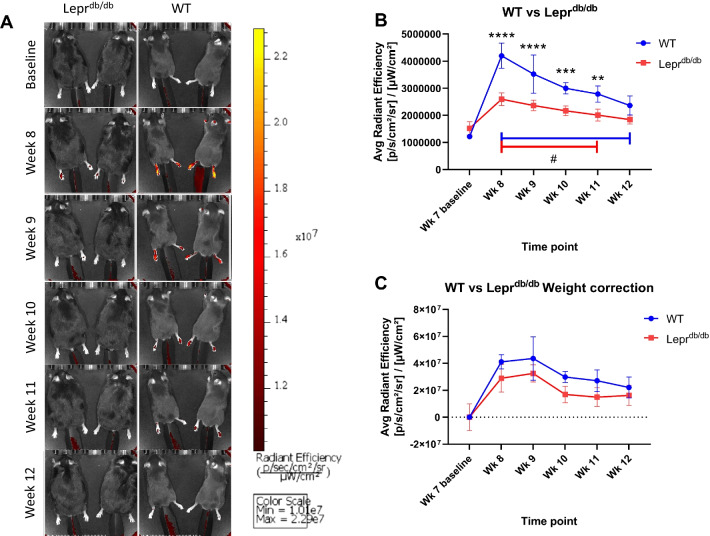
DiR signal is detectable in vivo using IVIS imaging. WT (*n* = 5) and Lepr^db/db^ (*n* = 5) mice were injected i.v. with 200 µl of PFC-NE. Images were taken using an IVIS Lumina system from 7 weeks of age (baseline) until 12 weeks of age (**A**). Injections were performed 72 h prior to the 8 week images. Fluorescence from the DiR component of the PFC-NE was detected within foot pads of each mouse and was tracked over time as Avg Radiant Efficiency ([p/s/cm^2^/sr]/[µW/cm^2^]) (**B**). A weight correction was performed to determine the extent to which differences seen between WT and Lepr^db/db^ mice were caused by differences in body mass (C). **(*p* < 0.01), ***(*p* < 0.001), ****(*p* < 0.0001). Error bar represents the standard deviation from five independent measurements

Two-Way ANOVA analysis of Weekly NIRF imaging of the foot pads of mice treated with PFC-NE showed there was a detectable DiR signal within the feet of both WT (Avg Radiant Efficiency [p/s/cm^2^/sr]/[µW/cm^2^]: Week 7: 1.22E + 06 ± 6.61E + 04; Week 8: 4.20E + 06 ± 4.57E + 05; Week 9: 3.53E + 06 ± 7.08E + 05; Week 10: 3.00E + 06 ± 2.11E + 05; Week 11: 2.79E + 06 ± 3.00E + 05; Week 12: 2.37E + 06 ± 3.52E + 05) and Lepr^db/db^ mice (Week 7: 1.52E + 06 ± 2.50E + 05; Week 8: 2.60E + 06 ± 2.35E + 05; Week 9: 2.37E + 06 ± 1.94E + 05; Week 10: 2.17E + 06 ± 1.80E + 05; Week 11: 2.01E + 06 ± 2.18E + 05; Week 12: 1.84E + 06 ± 1.61E + 05) (Fig. 4A). This signal was significantly higher than baseline readings and persisted for at least 3 weeks after injection in both WT (Week 7 vs 8: *p* = 0.0004; Week 7 vs 9: *p* = 0.0054; Week 7 vs 10: *p* = 0.0004; Week 7 vs 11: *p* = 0.0019; Week 7 vs 12: *p* = 0.0041) (Post hoc power analysis: Power = 1 for all time points) and Lepr^db/db^ groups (Week 7 vs 8: *p* = 0.0017; Week 7 vs 9: *p* = 0.0004; Week 7 vs 10: *p* = 0.0011; Week 7 vs 11: *p* = 0.0383; Week 7 vs 12: *p* = 0.0735) (Post hoc power analysis: Power ≥ 0.915 for all significant comparisons). However, it should also be noted that there was a significantly higher distribution of DiR signal to the feet of WT mice as compared to their Lepr^db/db^ counterparts (Fig. 4B) (Week 7: *p* = 0.6107; Week 8: *p* < 0.0001; Week 9: *p* < 0.0001; Week 10: *p* = 0.0009; Week 11: *p* = 0.0022; Week12: *p* = 0.0765) (Post hoc power analysis: Power ≥ 0.942 for all significant comparisons). To assess whether this difference may be due to the weight difference between groups, a weight correction was performed on the Average Radiant Efficiency data (Fig. 4C). After the correction there was no significant difference between the DiR signal in the WT and Lepr^db/db^ groups (Week 7: *p* > 0.9999; Week 8: *p* = 0.1314; Week 9: *p* = 0.2096; Week 10: *p* = 0.0935; Week 11: *p* = 0.1346; Week12: *p* = 0.8397) (see “Statistical analysis”). This suggests that the difference in fluorescence intensity between WT and Lepr^db/db^ is due at least in part to an effect of PFC-NE dosing per unit body mass. However, an inherent difference in phagocytic activity of macrophages between strains cannot be ruled out. To investigate further, we analyzed tissues and PFC-NE distribution using immunofluorescence.

### Immunofluorescence

#### Foot pad

**Fig. 5:**
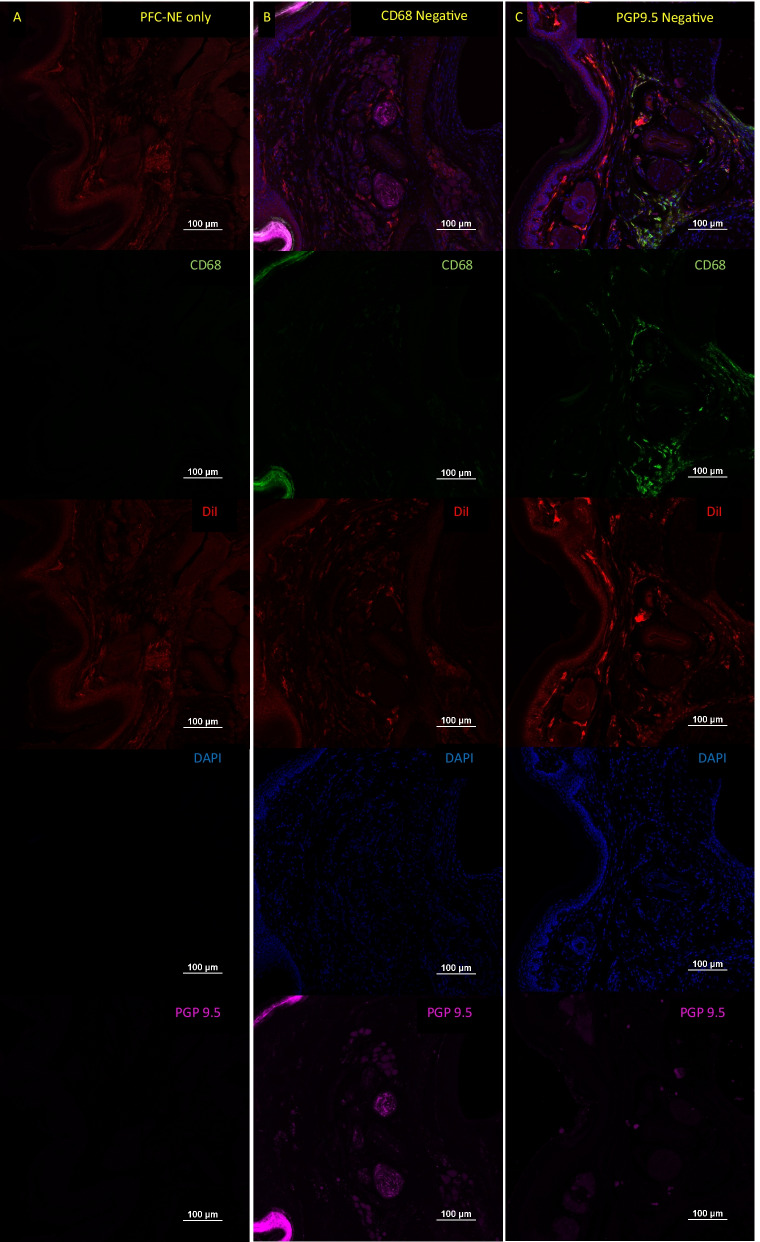
Negative Controls for Immunohistochemistry. To check the specificity of our histological staining procedures, and for the purpose of setting thresholds for analysis, control negative stains were performed on tissues sectioned from the feet of WT mice. To ensure the presence of DiI from PFC-NE, a slide from a WT PFC-NE injected mouse was hydrated 10 × in PBS, fixed for 10 min in 10% NBF, and coverslipped with Prolong Gold mounting medium (**A**). For CD68 (**B**) and PGP9.5 (**C**) control negative slides, the staining procedure was the same as all other stains except for the omission of the primary antibody in question. Scale bar = 100 µm

**Fig. 6:**
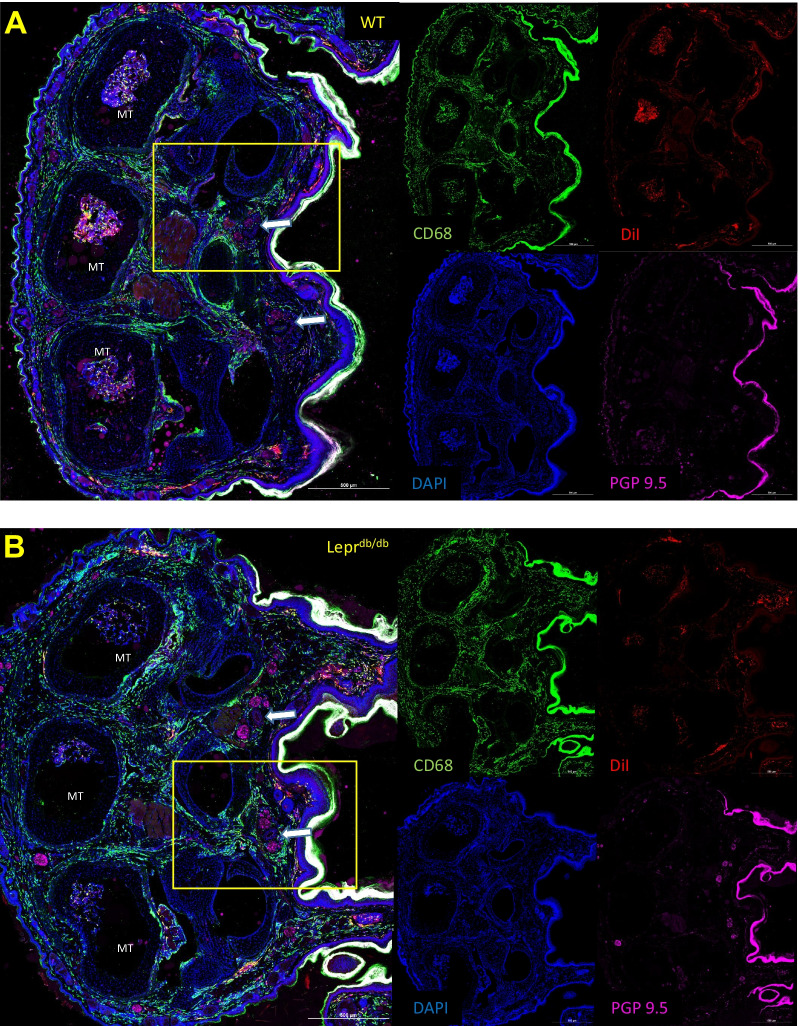
Identification CD68^+^ and DiI^+^ cells within the hind paws of WT and Lepr^db/db^ mice. Hind foot histological sections of WT (**A**) and Lepr^db/db^ (**B**) mice injected with PFC-NE 72 h prior to tissue collection were subsequently stained with CD68 and PGP9.5 antibodies (and DAPI) and reviewed for the distribution of CD68^+^ DiI^+^ double positive cells. DiI from the PFC-NE was found within CD68^+^ cells in dermis, hypodermis, bone marrow, and around the neurovascular bundles. Images were taken at 20 × and a composite image was formed using the tile scan feature of the Nikon confocal microscope. Yellow rectangles represent the area of focus around the neurovascular bundles (white arrows) for Fig. 6. Scale bar = 500 µm, *MT* metatarsals

**Fig. 7:**
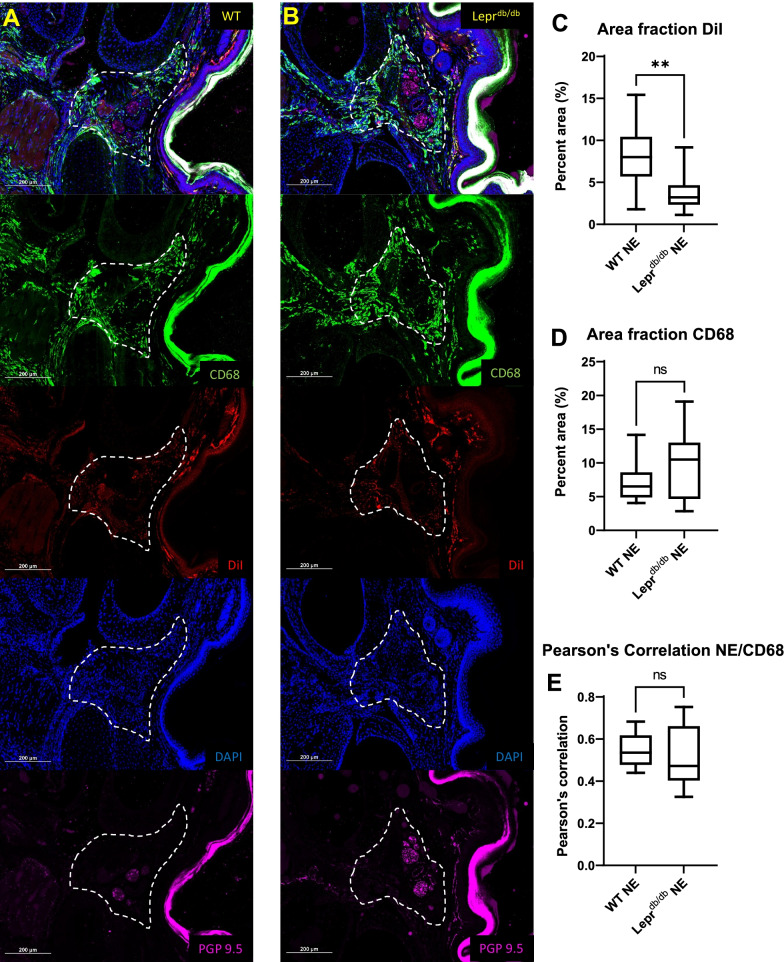
Quantification of CD68 and DiI signal in hypodermal tissue surrounding the Neurovascular bundles of the hind limb foot pad. Analysis of the hypodermal regions (dotted white line) between metatarsals 2, 3 and 4 from hind limb foot pads of WT (*n* = 3) (**A**) and Lepr^db/db^ (*n* = 3) (**B**) mice showed a significantly lower amount of DiI surrounding the nerves (N) of neurovascular bundles of Lepr^db/db^ mice as compared to WT mice (*p* = 0.0063) (**C**). No significant difference was found for percent area of CD68 (*p* = 0.3389) (**D**) or Pearson’s correlation for CD68 and DiI (*p* = 0.7317) (**E**) when comparing WT and Lepr^db/db^ mice. Scale bar = 200 µm. Error bar represents the standard deviation from twelve independent measurements

**Fig. 8:**
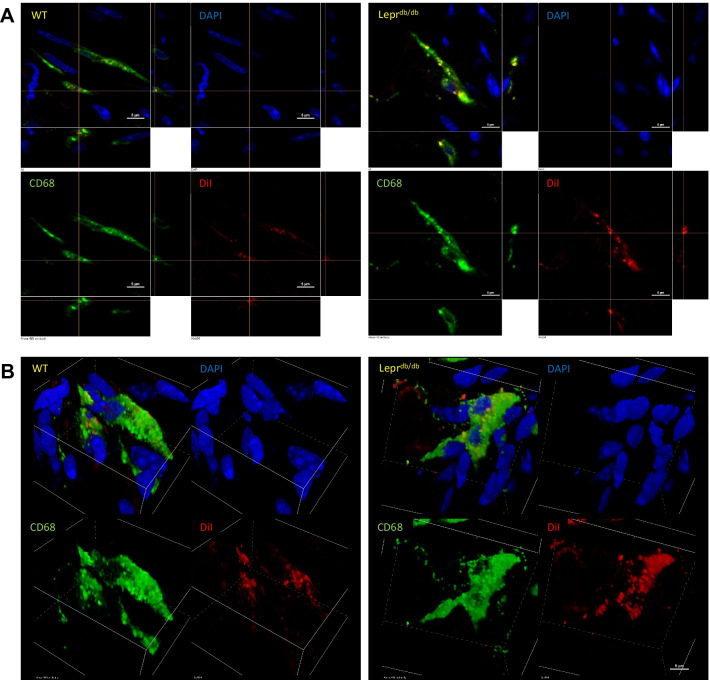
High magnification images of CD68^+^DiI^+^ double positive cells from hypodermal tissues of the foot in WT and Lepr^db/db^ mice. Individual CD68^+^DiI^+^ double positive cells were identified in the hypodermis of both WT and Lepr^db/db^ mice, and high-resolution images were taken using a 100 × objective to confirm colocalization of DiI and CD68 signal within these cells (**A**). 3D renderings of these confocal images were also generated to demonstrate that the DiI signal was within the CD68^+^ cells (**B**). Scale bar = 5 µm

Distribution of PFC-NE into macrophages occurs in most tissues of the hind foot including the dermis, hypodermis, bone marrow, and around the neurovascular bundles (Figs. 5, 6). Interestingly, one of the only tissues in which we could not detect a DiI signal from the PFC-NE was the nerves that run in the neurovascular bundles between the metatarsals. These nerves contained very few CD68^+^ cells, and when CD68^+^ cells were detectable in a histological section, they did not contain a DiI signal. Nested T-test analysis of percent area measurements for CD68 (WT: 7.066 ± 2.799; Lepr^db/db^: 9.890 ± 5.192) and DiI (WT: 8.452 ± 3.702; Lepr^db/db^: 3.752 ± 2.350) signals within hypodermal region surrounding the neurovascular bundle of the foot pad showed that percent area for DiI was significantly lower in Lepr^db/db^ mice as compared to the WT mice (Fig. 7A–C) (*p* = 0.0063) (Post hoc power analysis: Power = 0.96). These results reflect our in vivo imaging data for DiR fluorescence in the foot of WT and Lepr^db/db^ mice (Fig. 4). No significant difference was seen in the percent area of CD68 signal between WT and Lepr^db/db^ mice (Fig. 7D; *p* = 0.3389). Analysis of the distribution of DiI and CD68 within this region also showed no significant difference in Pearson’s correlation between WT (0.5496 ± 0.08095) and Lepr^db/db^ mice (0.5161 ± 0.1431) (*p* = 0.7317), which suggests that colocalization of PFC-NE to CD68^+^ cells is similar between groups (Fig. 7E). High resolution 100 × magnification images were also taken to show the colocalization of the DiI signal from our PFC-NE to CD68^+^ cells within the hypodermis of the foot (Fig. 8).

#### Sciatic nerve

**Fig. 9:**
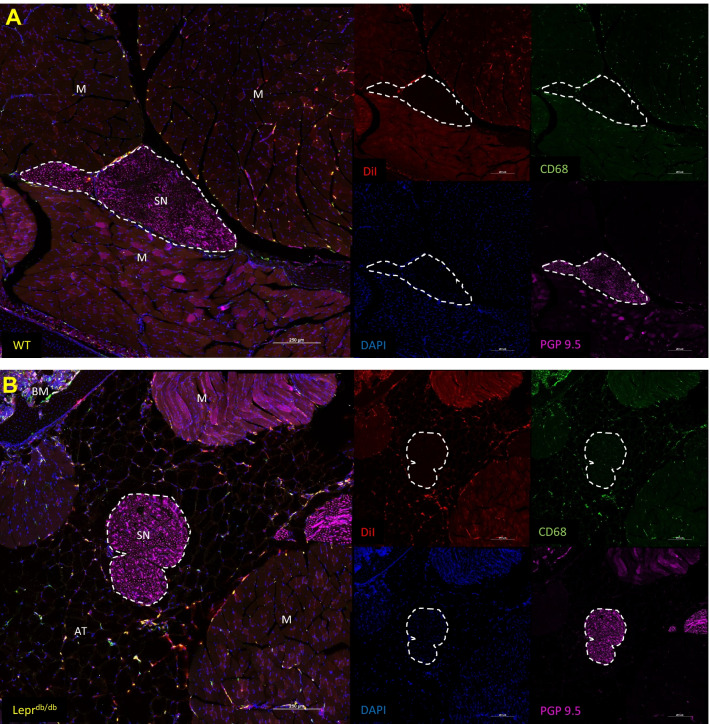
Identification CD68^+^ and DiI^+^ cells within the thighs of WT and Lepr^db/db^ mice. Histological sections of the left and right thighs from WT (**A**) and Lepr^db/db^ (**B**) mice injected with PFC-NE 72 h prior to tissue collection were subsequently stained with CD68, PGP9.5 and DAPI and reviewed for the distribution of CD68^+^DiI^+^ double positive cells. DiI from the NE was found within CD68^+^ cells in bone marrow (BM), perineural adipose tissue (AT), muscle (M), and sciatic nerve (SN; dotted white line). Images were taken at 20 × and a composite image was formed using the tile scan feature of the Nikon confocal microscope. Scale bar = 250 µm

**Fig. 10:**
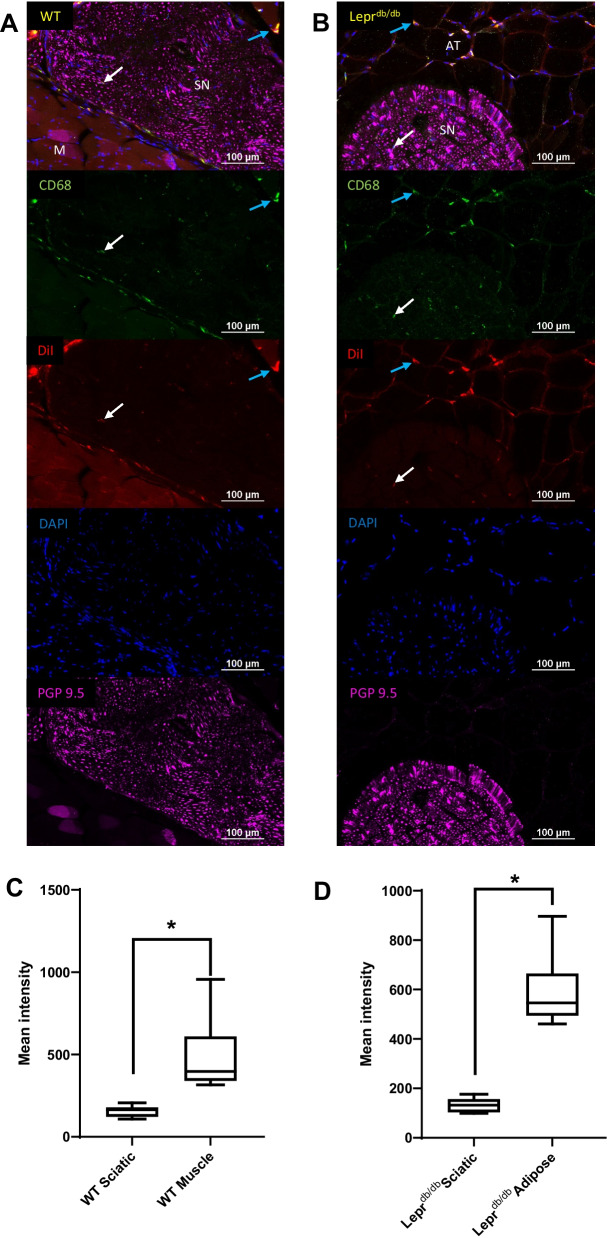
Quantification of CD68^+^ and DiI^+^ cells within the sciatic nerves of WT and Lepr^db/db^ mice. A closer examination of DiI present within the sciatic nerves (SN) of both WT (**A**) and Lepr^db/db^ (**B**) mice was performed to determine if sciatic nerves showed a lower intensity of DiI in CD68^+^ cells as compared to the surrounding tissues. Three CD68^+^DiI^+^ double positive cells were identified from the left and right sciatic nerves (*n* = 6) of each WT and Lepr^db/db^ mouse for comparison with three cells from adjacent tissues [muscle (M) (*n* = 6) or adipose (AT) (*n* = 6)] on the same slide (**C**, **D**). This comparison revealed a significantly lower mean intensity of DiI signal within the CD68^+^ cells of the sciatic nerve from WT (*p* = 0.0313) and Lepr^db/db^ (*p* = 0.0313) mice as compared to muscle and adipose tissue respectively. CD68^+^NE^+^ double positive cells are identified in the sciatic nerve (white arrows) and peripheral tissue (blue arrows) of each image. Scale bar = 100 µm. Error bar represents the standard deviation from six independent measurements

Tissue sections of the thighs from WT and Lepr^db/db^ mice showed distribution of the PFC-NE into the macrophages of every tissue type including muscle, adipose, bone marrow, and sciatic nerve (Fig. 9A, B); however a Wilcoxon matched-pairs signed rank test showed that the DiI signal observed in the sciatic nerves (WT: Mea*n* = 156.5, IQR = 57.9; Lepr^db/db^: Mea*n* = 132.4, IQR = 52.8) was significantly weaker as compared to the surrounding tissues (WT Muscle: Mea*n* = 485.2, IQR = 269.4; Lepr^db/db^ Adipose: 590.4, IQR = 170.4) in both the WT (*p* = 0.0313) (Post hoc power analysis: Power = 0.912) and Lepr^db/db^ (*p* = 0.0313) (Post hoc power analysis: Power = 1) groups (Fig. 10A–D). The sciatic nerves of Lepr^db/db^ mice were consistently surrounded by a large amount of adipose tissue which contained hypertrophic adipocytes and CD68^+^DiI^+^ macrophages, which may explain the NIRF imaging results showing significant difference between WT and Lepr^db/db^ mice. Enhanced adipose tissue macrophage activity is likely drawing uptake of PFC-NE into these tissues as opposed to paws where we focused our investigation. By contrast, the perineural fat pads of WT mice were harder to identify by fluorescence microscopy due to their relatively small size.

#### Lumbar spinal cord and DRGs

**Fig. 11:**
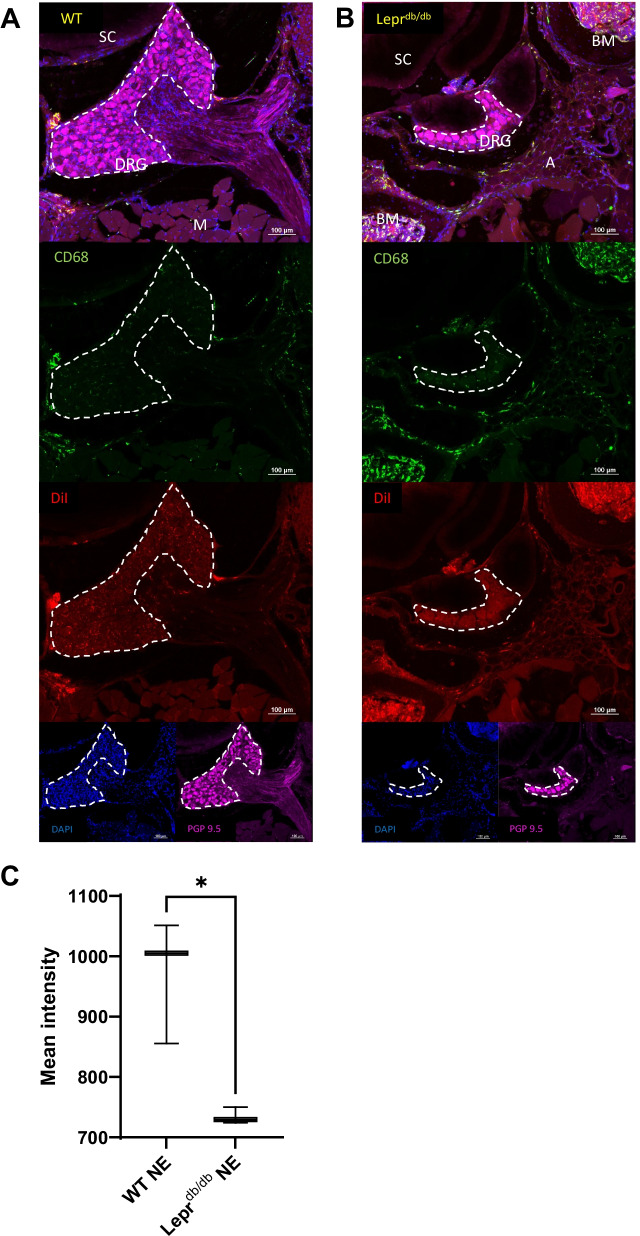
Identification CD68^+^ and DiI^+^ cells within the lumbar DRGs of WT and Lepr^db/db^ mice. Sections containing lumbar DRG tissue from WT (**A**) and Lepr^db/db^ mice (**B**) were reviewed to determine the location of CD68^+^DiI^+^ cells. CD68^+^DiI^+^ double positive cells were identified within the DRG, perineural adipose tissue, and meninges surrounding the DRGs. Analysis of the DiI mean signal intensity within the DRG itself revealed a significant difference between WT (*n* = 3; **C**) and Lepr^db/db^ (*n* = 3; **D**) mice (*p* = 0.0167). Images were taken at 20 × and a composite image was formed using the tile scan feature of the Nikon confocal microscope. Scale bar = 100 µm. Error bar represents the standard deviation from three independent measurements

**Fig. 12:**
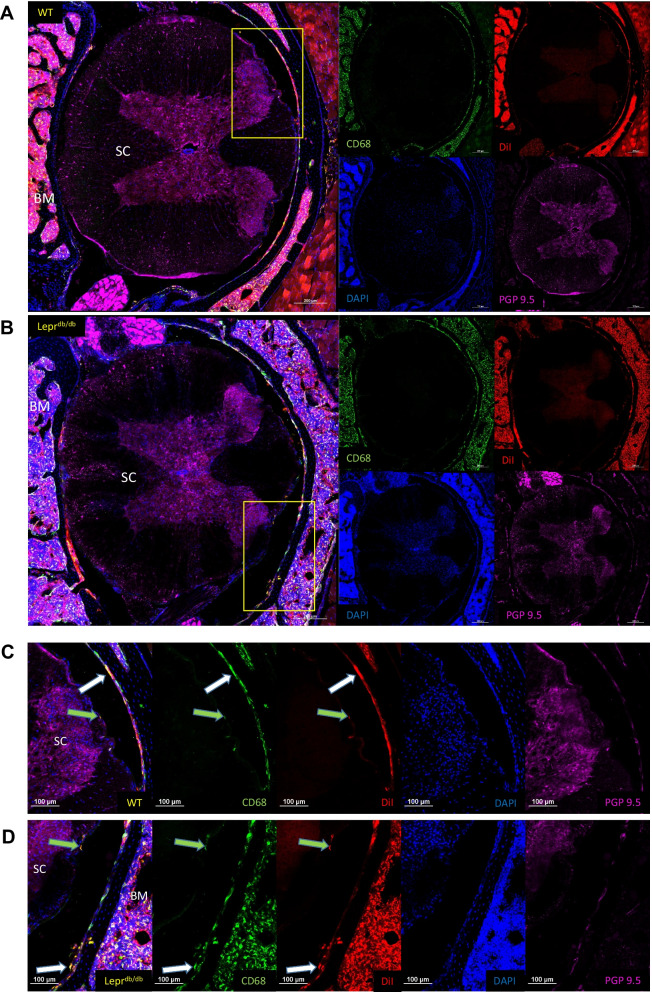
Identification CD68^+^ and DiI^+^ cells within the spinal column of WT and Lepr^db/db^ mice. Sections containing lumbar spinal cord tissue from WT (**A**) and Lepr^db/db^ mice (**B**) were reviewed to determine the location of CD68^+^DiI^+^ cells. CD68^+^DiI^+^ double positive cells were identified within the meninges and bone marrow, but not in white matter, grey matter, or nerve roots of the spinal cord. Closer inspection of the meninges revealed CD68^+^DiI^+^ double positive cells within the dura/arachnoid mater (white arrows) and pia mater (green arrows) (**C**, **D**). Images were taken at 20 × and a composite image was formed using the tile scan feature of the Nikon confocal microscope. Scale bar = 200 µm (**A**, **B**); 100 µm (**C**, **D**)

Transverse sectioning the entire spinal column allowed us to preserve the bone marrow within the vertebrae, as well as the meninges surrounding the DRGs (Fig. 11) and spinal cords (Fig. 12). DiI from PFC-NE was detectable in CD68^+^ cells within the bone marrow, DRG, meninges, and adipose tissue which lay adjacent to the DRGs of Lepr^db/db^ mice. Interestingly, a *T*-test showed the mean intensity of the DiI signal observed within the DRGs of Lepr^db/db^ mice (734.6 ± 13.82) was significantly lower as compared to that in the WT mice (970.5 ± 102.3) (*p* = 0.0167) (Post hoc power analysis: Power = 0.977) (Fig. 11C, D). No DiI signal was observed within the parenchyma of the spinal cord, which would suggest that the PFC-NE is unable to penetrate the blood–brain barrier (BBB) in these models.

## Discussion

With T2DM becoming a larger burden on public health systems, and the high prevalence of DPN within T2DM patients, it is important to understand not only the mechanisms behind DPN, but also how we might effectively combat its progression. The aim of this study was to explore the potential of a new PFC-NE based imaging agent that has both the ability to effectively target macrophages, which have been shown to play a major role in neuropathic pain, and to label these macrophages for further observation by multiple imaging modalities [10, 11, 14–16]. To do this we developed and characterized a PFC-NE which contains two fluorescent dyes (DiI and DiR) to track its tissue distribution along the sensory pathway of the hind paw to see what potential this PFC-NE holds as a therapeutic option for DPN.

The major advantage that comes from incorporation of DiR into this PFC-NE is the ability to track its distribution in vivo. Using non-invasive NIRF imaging we were able to detect significant increases in DiR signal from the foot pads of WT and Lepr^db/db^ mice as compared to baseline measurements. We also found that Lepr^db/db^ mice had significantly lower distribution of the PFC-NE to their foot pads as compared to WT mice. This was somewhat surprising, as we hypothesized that Lepr^db/db^ mice would have a higher concentration of activated macrophages in the tissues of the distal limb and increased DiR signal from PFC-NE uptake into these macrophages. This was also based on previous studies in other inflammatory pain states which displayed increased NIRF signal [28, 36]. Upon histological examination of these tissues we were able to determine that, despite a moderate increase in the CD68^+^ signal in the hypodermal region surrounding the neurovascular bundles of the feet of Lepr^db/db^ mice, there was a significant decrease in the DiI signal from PFC-NE present within this same region. However, since there was not a corresponding decrease in the Pearson’s correlation between the two signals, the decrease in percent area that was observed likely represents a decrease in the amount of PFC-NE that was taken into each cell, and not a decrease in the percentage of CD68^+^ cells which contain PFC-NE. In previously published in vitro experiments we have shown that nanoemulsion uptake is dependent on the concentration of nanoemulsion to which macrophages are exposed, the length of time that they are exposed, and the ability of macrophages to phagocytize [28]. Earlier studies also showed stable in vivo macrophage labeling with PFC-NEs upon i.v. injection in multiple rodent models [27, 28, 36, 38, 39, 43]. Considering this and the fact that the histological tissues were all processed 72 h after injection, we believe there are two potential explanations for the detectable decrease in DiI signal. One is that there is a fundamental difference in the phagocytic ability of macrophages from diabetic mice that would reduce their ability to take in the PFC-NE. This line of reasoning is supported by previous studies which showed phagocytic activity is decreased in human peripheral blood mononuclear cells from T2DM patients and murine macrophages cultured under high glucose conditions [44, 45]. Future studies will assess phagocytic ability in the foot pad macrophages of Lepr^db/db^ mice, since this may represent a decrease in the ability to treat these macrophages with PFC-NE based therapies. A second possibility is that the PFC-NE is preferentially deposited in different tissues in diabetic mice, thus reducing the availability of PFC-NE to the tissue macrophages of the foot. Given the macrophage specific nature of our PFC-NE and of similar PFC-NEs from previous publications [27, 28, 36], substantial uptake of PFC-NE by cell types other than macrophages appears unlikely. Instead, the large population of adipose tissue macrophages (ATMs) in Lepr^db/db^ mice represent a more likely explanation. Upon examination of various tissues for DiI distribution, it became clear that there was a significant amount of PFC-NE being taken into the ATMs. Based on this observation we performed a weight correction on the foot pad DiR signal, which eliminated any significant differences seen between the two groups of mice. These findings are important because it shows that ATMs are a major point of uptake of this PFC-NE, and that weight may need to be accounted for in delivery of this and similar agents in future.

Images of the neurovascular bundles of the foot revealed that PFC-NE was not detectable within the larger nerves of either the WT or Lepr^db/db^ mice. A similar difference to that seen in the peripheral nerves of the neurovascular bundles was also seen in the sciatic nerve. The CD68^+^ cell within the sciatic nerves of both WT and Lepr^db/db^ mice have lower levels of DiI than the surrounding tissues. It is unclear why the macrophages within the sciatic and distal nerves of the foot would have a reduced level of PFC-NE uptake. One potential explanation for this is related to the Blood-Nerve Barrier (BNB), which limits diffusion of substances into the peripheral nerves in a similar manner to the BBB [46]. In previous studies BNB disruption has been shown in patients with diabetes [47] and Lepr^db/db^ mice [48], however it is possible that in the current study the BNB is still intact and PFC-NE is hindered from entering the sciatic nerve. This suggests that these macrophages may not respond as effectively to PFC-NE treatments as compared to other macrophages when the BNB is intact. This is supported by the similar levels of DiI found in both WT and Lepr^db/db^ sciatic nerves. Further studies are needed to examine the function of the BNB in older Lepr^db/db^ mice to determine if PFC-NE would eventually increase in the sciatic nerve macrophages. Another notable difference between the WT and Lepr^db/db^ sciatic nerves was the large accumulation of hypertrophic adipocytes visible around the sciatic nerves of Lepr^db/db^ mice. The inflammatory potential of the ATMs within this adipose tissue may have some effect on the sciatic nerve through the secretion of cytokines, and thus may be a more viable target for DPN than sciatic nerve macrophages. This is supported by a previous study in which perineural administration of both saporin-conjugated anti-Mac1 antibodies and TC-25594-[(5-ethoxy-3-pyridinyl)-N-methyl-(3E)-3-buten-1-amine difumarate], an nAChR agonist capable of inhibiting macrophages, reduced neuropathic pain in a high fat diet T2DM model [14]. While this study was not specifically aiming to treat ATMs, the perineural administration of these treatments would have likely encompassed them, providing a strong rationale for the benefits of specifically targeting perineural ATMs.

Examination of DRG sections showed uptake of PFC-NE within CD68^+^ cells of the DRGs. This is a highly significant finding since DRGs represent a microenvironment in which the somata of primary sensory neurons are in direct contact with macrophages that can be targeted using this PFC-NE. Given that a previous study has already established that treatment of DRG macrophages has the ability to reduce hypersensitivity and loss of IENFs in a chemotherapy induced peripheral neuropathy model, we believe that the presence of our PFC-NE within these macrophages strongly suggests that PFC-NE therapies would have beneficial outcomes in DPN [49]. As was noted perineurally in the sciatic nerve, deposits of hypertrophic adipose tissue were also seen immediately adjacent to the DRGs of Lepr^db/db^ mice where there was a large reservoir of CD68^+^DiI^+^ double positive cells. The relatively lower intensity of the DiI deposits within the DRG of Lepr^db/db^ mice as compared to WT mice reinforces our previous postulation that PFC-NE preferentially deposits in the CD68^+^ cells of adipose tissue as compared to other tissue, and in this case (as it was in the foot) the DRGs seem to receive less PFC-NE as a result.

The survey taken of the lumbar region of the spinal cord showed a lack of penetration of the PFC-NE into the parenchyma of the spinal cord. This suggests that the PFC-NE was not able to penetrate the BBB. Despite this, CD68^+^/DiI^+^ double positive cells were observable in all three layers of the meninges, and as we saw in earlier sections of the femur and metatarsals, we observed PFC-NE within the bone marrow of the vertebral column. While the observation of our PFC-NE within the meninges of WT and Lepr^db/db^ mice may not be of direct significance to this current study, it does represent an important finding that could be of potential benefit in cases of meningitis, where the specific targeting of macrophages could quiet potentially dangerous inflammatory responses. More work needs to be done in order to determine if these meningeal macrophages represent a resident population that can take up PFC-NE from the circulation, or if they are macrophages that have migrated from the peripheral circulation to the meninges after phagocytizing PFC-NE.

It is known that the production of adipokines, cytokines and free fatty acids by hypertrophic adipocytes causes recruitment and activation of macrophages which contribute to the development of insulin resistance [50–52]. Moreover, IL-6, IL-1β, and TNFα are all produced by hypertrophic adipocytes and M1 macrophages, and all three of these cytokines have been implicated in hyperalgesia [10, 50]. TNFα may also play a critical role in insulin resistance, specifically by downregulation of GLUT-4 and IRS-1 [53–55]. Based on the uptake of our PFC-NE within the ATMs of Lepr^db/db^ mice and on previous studies showing the effectiveness of similarly designed PFC-NE loaded with celecoxib at reducing macrophage-driven inflammation we believe that our novel PFC-NE agent will be equally effective if loaded with treatments targeted toward alteration of macrophage function and/or secretory output. This would have the potential to reduce downstream production of inflammatory cytokines, such as TNF-α, IL-1β, and IL-6, or potentially skew the phenotype of ATMs to a more anti-inflammatory phenotype, which may represent the more natural state of macrophages in adipose tissue [36, 52, 54, 56]. This would not only result in reducing production of these cytokines in adipose tissue adjacent to the various portions of the sensory apparatus that we have assessed in this study, but may also reduce the systemic insulin resistance seen in T2DM patients. This in turn would have the potential to reduce hyperglycemia, another cause of DPN in T2DM [2].

## Conclusions

Based on the results obtained from this study, we can conclude that the novel PFC-NE-based imaging agent we have developed can effectively target macrophages in the Lepr^db/db^ model of T2DM. We further hypothesize that drug loading of the PFC-NE particles with macrophage targeting treatments, such as celecoxib, has the potential to reduce inflammation in ATM both systemically and perineurally to reduce DPN and insulin resistance in this model of T2DM. Future studies with this PFC-NE will aim to test this hypothesis by examining markers of insulin resistance and neuropathic pain.

## Data Availability

The datasets used and analyzed during the current study are available from the corresponding author on reasonable request.

## Abbreviations

°C: Degrees celsius
ANOVA: Analysis of variance
ATCC: American type culture collection
ATM: Adipose tissue macrophages
BBB: Blood–brain barrier
BNB: Blood-nerve barrier
CNS: Central nervous system
CO_2_: Carbon dioxide
DAPI: 4′,6-Diamidino-2-phenylindole
DiI/DiIC18(3): 1,1′-Dioctadecyl-3,3,3′,3′-tetramethylindocarbocyanine perchlorate
DiR: 1,1′-Dioctadecyl-3,3,3′,3′-tetramethylindotricarbocyanine Iodide
DLS: Dynamic light scattering
DMSO: Dimethyl sulfoxide
DPN: Diabetic peripheral neuropathy
DRG: Dorsal root ganglia
EDTA: Ethylenediaminetetraacetic acid
e.g.: Exempli gratia/for example
GLUT-4: Glucose transporter 4
HC: Hydrocarbon
HeNe: Helium–neon
IENF: Intraepidermal nerve fiber
IRS-1: Insulin receptor substrate-1
λ: Wavelength
μM: Micromolar
μm: Micrometer
µW/cm^2^: Microwatts per square centimeter
mL: Milliliters
MRI: Magnetic resonance imaging
NBF: Neutral buffered formalin
NIRF: Near infra-red fluorescence
nm: Nanometers
OCT: Optimum cutting temperature
PGP9.5: Protein gene product 9.5
p/s/cm^2^/sr: Photons per second per square centimeter per steradian
PBS: Phosphate-buffered saline
PFA: Paraformaldehyde
PFC-NE: Perfluorocarbon nanoemulsion
RT: Room temperature
SNRI/SSRI: Selective norepinephrine/serotonin reuptake inhibitor
T2DM: Type 2 diabetes mellitus
v/v: Volume/volume
WT: Wild-type
w/w: Weight/weight
